# Prosocial Orientation Alters Network Dynamics and Fosters Cooperation

**DOI:** 10.1038/s41598-017-00265-x

**Published:** 2017-03-23

**Authors:** David Melamed, Brent Simpson, Ashley Harrell

**Affiliations:** 1 Department of Sociology, The Ohio State University, Columbus, OH 43210 United States; 2 Department of Sociology, University of South Carolina, Columbia, SC 29208 United States; 3 Organizational Studies, University of Michigan, Ann Arbor, MI 48109 United States

## Abstract

Dynamic networks have been shown to increase cooperation, but prior findings are compatible with two different mechanisms for the evolution of cooperation. It may be that dynamic networks promote cooperation even in networks composed entirely of egoists, who strategically cooperate to attract and maintain profitable interaction partners. Alternatively, drawing on recent insights into heterogeneous social preferences, we expect that dynamic networks will increase cooperation only when nodes are occupied by persons with more prosocial preferences, who tend to attract and keep more cooperative partners relative to egoists. Our experiment used a standard procedure to classify participants *a priori* as egoistic or prosocial and then embedded them in homogeneous networks of all prosocials or all egoists, or in heterogeneous networks (50/50). Participants then interacted in repeated prisoner's dilemma games with alters in both static and dynamic networks. In both heterogeneous and homogeneous networks, we find dynamic networks only promote cooperation among prosocials. Resulting from their greater cooperation, prosocials’ relations are more stable, yielding substantially higher fitness compared to egoists in both heterogeneous and homogeneous dynamic networks. Our results suggest that a key to the evolution and stability of cooperation is the ability of those with prosocial preferences to alter their networks.

## Introduction

Understanding the conditions that promote other-regarding motivations and cooperative behaviors are fundamental puzzles for the social and biological sciences^1–5^. Recent work has focused on structured or networked population as a key mechanism in the evolution of cooperation. In particular, dynamic networks, or networks where ties can be formed and/or broken, generate cooperation by allowing individuals to shed ties to non-cooperators or defectors^6–11^. In turn, defectors may alter their strategies to retain their remaining ties. In these networks, cooperation diminishes through time^8^, but remains a more viable strategy than in static networks.

Existing findings are compatible with two different explanations of why and when dynamic networks promote cooperation. First, dynamic networks might increase cooperation even in networks composed entirely of egoists, who strategically cooperate in order to attract and maintain profitable interaction partners^12, 13^. But we expected that dynamic networks increase cooperation mainly when nodes are occupied by those with more prosocial preferences^14–16^: because they are more cooperative, prosocials^17, 18^ will tend to attract and keep more cooperative partners relative to those with egoistic preferences. These two accounts have very different implications for the evolution of prosocial preferences and behaviors. But prior work on dynamic networks ignores insights from the literature on social values, either assuming that all actors are egoistic, or inferring “types” (prosocial versus egoistic) from the behaviors that those dispositions are supposed to explain (cooperation versus non-cooperation). As a consequence, previous work cannot discriminate between the two accounts of when and why dynamic networks promote cooperation.

Our experiments included a total of 360 participants interacting in twelve-person networks. Before the experiment took place (average of 26.4 days), we measured prosocial versus egoistic dispositions *a priori* using a standard procedure for measuring social values^17, 19^. We used these measures to sort participants into homogeneous networks of all prosocials or all egoists, or heterogeneous networks, comprised of 50% prosocials and 50% egoists. Each network of 12 completed both a static and dynamic condition, in random order, for a total of 60 networks (see *SI Appendix*). Network ties represented an opportunity to interact in a prisoner's dilemma (PD). We predicted that prosocials would keep their ties longer as a result of their higher levels of cooperation, which would result in increased earnings. Moreover, in dynamic networks, we expected greater inequality in heterogeneous (versus homogeneous) networks, with that inequality favoring prosocials.

For both the static and dynamic phases, there were 12 rounds of interaction in the PD that entailed choosing whether to cooperate or defect with each alter. Participants were not told how many rounds there would be. Prior studies typically force participants to either cooperate or defect with all of their alters^7, 8, 20^, but in the interest of realism and consistent with findings that participants use conditional responses in PD games^21–24^, we allowed them to make independent decisions for each relation. The incentive structure for the PD followed similar studies^8^. Mutual cooperation resulted in +4 points each; mutual defection resulted in +1 point each; and unilateral defection and unilateral cooperation resulted in +7 and −1 points each, respectively.

Each participant initially had three ties. In static networks, these ties did not change. In dynamic networks, participants were asked whether they wanted to drop an alter after each round. If so, they were able to pick which tie to unilaterally delete. Following prior work, ties were not replaced for participants who were dropped^6, 7, 20^. Participants who dropped an alter were given a new tie to a random new alter with whom they had not previously interacted. In principle, new ties can be formed based on reputations^25^, simply at random, or at random from some subpopulation^9^. Prior work on dynamic networks^7, 8, 20^ gives participants seeking new ties reputational information on prospective alters. But here we sought to isolate the effects of network dynamics by not giving participants any reputational information on prospective partners. Either of the random mechanisms would allow us isolate the effects of network dynamics, but we limited connections to those who had not previously interacted. The fact that participants could not reconnect to previously severed ties (or to those who had severed ties to them) is common in the real-world. For instance, we do not typically remarry those we have divorced; similarly, we do not generally reestablish business partnerships we have severed due to the partner’s untrustworthiness, etc.

## Results

Among prosocials, dynamic networks promote cooperation in both *homogeneous* networks (b = 0.98, *p* < 0.01) and *heterogeneous* networks (b = 1.01, *p* < 0.05; unless otherwise noted, all *p*-values determined using (generalized) linear mixed models accounting for the nested structure of the data (*SI Appendix*)). Among egoists, the effect of dynamic networks is not significantly different from zero in either *homogeneous* (b = 0.65, *p* = n.s.) or *heterogeneous* networks (b = 0.603, *p* = n.s.). Only when comparing homogeneous static to heterogeneous dynamic networks do we find an increase in cooperation among egoists (b = 0.96, *p* < 0.05).

**Figure 1:**
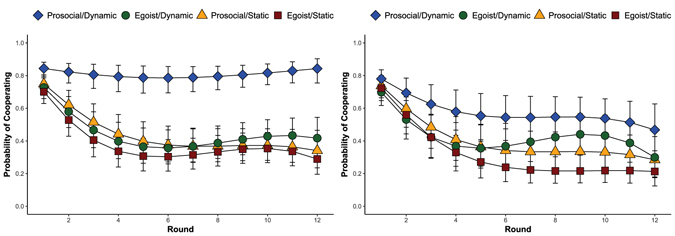
Marginal probability of cooperation with each alter in (**A**) homogeneous and (**B**) heterogeneous networks. Error bars refer to 95% confidence intervals around the marginal means.

To show how these processes vary with time, Fig. 1A and B plot the marginal probabilities of cooperating across the 12 rounds in the homogeneous (1A) and heterogeneous (1B) networks. Beginning with the homogeneous networks, Round 1 shows no differences in cooperation rates. But even in round 2, prosocials in homogeneous dynamic networks cooperate at higher levels than all other groups (*p* < 0.05). Further, whereas all other groups exhibit the characteristic decline in cooperation through time^3^, prosocials in homogeneous dynamic networks cooperate at rates heretofore unseen in this literature. Figure 1B shows similar patterns for heterogeneous networks, except that the mixing reduces cooperation among prosocials and increases cooperation among egoists, yielding less pronounced differences between the two. Together these findings suggest that whether dynamic networks promote cooperation depends crucially on the presence of prosocials.

A conditional logistic regression shows that participants were less likely to drop alters who cooperated (15%) over those who defected (41%, *p* < 0.001). As prosocials are more cooperative, this translates into more durable relationships between them. In both homogeneous and heterogeneous networks, prosocials’ relationships lasted an average of almost one round longer (b = 0.97, *p* < 0.001) than egoists’ relationships. Importantly, we find that the number of times participants cooperated with each alter explains the difference between egoists’ and prosocials’ relationship duration. Each time participants cooperated with an alter, that relationship lasted 0.91 rounds longer (*p* < 0.001), on average, and when this term is included, the effect of participants’ prosociality becomes indistinguishable from zero (b = −0.26, *p* = n.s.).

**Figure 2:**
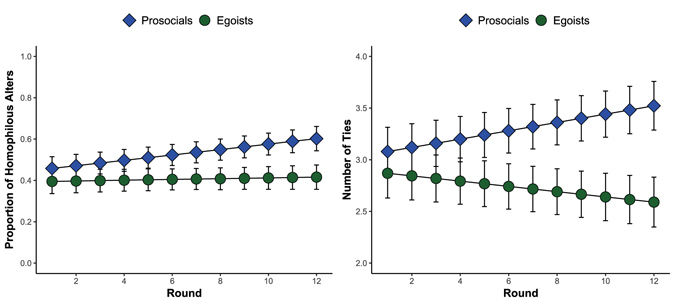
Marginal (**A**) homophily (proportion of alters that have the same Social Value Orientation as ego) and (**B**) number of ties (the count of ties for each participant) in heterogeneous dynamic networks. In terms of number of ties, each participant began with three ties but marginal means are shown. Error bars refer to 95% confidence intervals around the marginal means.

One implication of the greater stability of prosocials’ ties is that their networks become more homophilous^26–28^ over time, as shown in Fig. 2A. Specifically, prosocials’ networks were 15.6% more homophilous by the end of the experiment (*p* < 0.001). Recall that those who dropped alters were randomly connected to new alters, whereas the ties of those who were dropped by others were not replaced. We found that prosocials acquired more ties than egoists in heterogeneous dynamic networks, as shown in Fig. 2B. By the end of the study, egoists had *lost* an average of 0.36 ties, whereas prosocials had gained an average of 0.48 ties (both *p* < 0.01). These findings on homophily and number of ties are striking given that participants were not allowed to select their partners based, e.g., on reputations. Instead, partners were assigned at random. Thus, homophily and tie frequency emerged simply as a result of selective shedding of unwanted ties.

**Figure 3:**
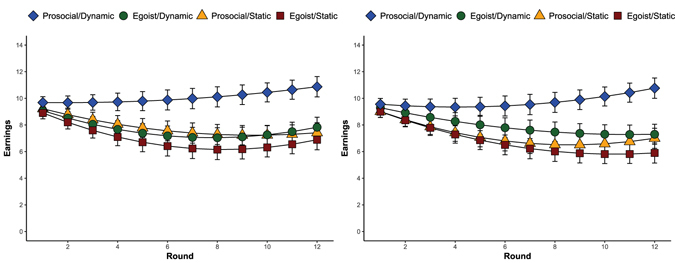
Marginal earnings/fitness in (**A**) homogeneous and (**B**) heterogeneous networks. Error bars refer to 95% confidence intervals around the marginal means.

We now assess how the outcomes reported thus far impact earnings. Dynamic networks do not significantly increase the earnings of egoists in either homogeneous (b = 0.97, *p* = n.s.) or heterogeneous (b = 0.89, *p = *n.s.) networks. By contrast, prosocials in dynamic networks – either homogeneous (b = 3.96, *p* < 0.001) or heterogeneous (b = 2.18, *p* < 0.001) – earn substantially more. Figure 3A and B show marginal earnings over time. Mediation analyses demonstrate that the average duration of relationships in a given round explains the effect of prosociality on earnings in both homogeneous and heterogeneous dynamic networks. For each round increase in average relation duration, prosocials in dynamic networks earn 0.46 additional points (*p* < 0.001), while the effect of duration is insignificant for egoists (b = 0.11, *p* = n.s.).

**Figure 4:**
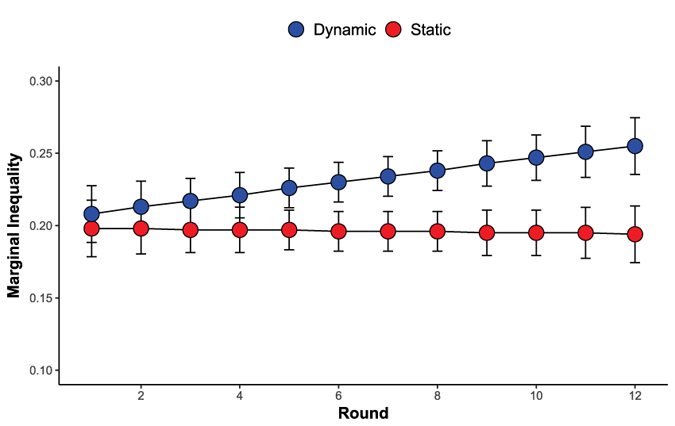
Marginal inequality (Gini coefficients) in the heterogeneous networks. Error bars refer to 95% confidence intervals around the marginal means.

Following recent work^29^, we also investigated how different networks lead to inequality in earnings over time. Based on the patterns reported thus far, we expected that, in heterogeneous populations, dynamic networks would promote greater inequality, favoring prosocials. Figure 4 gives marginal inequality (Gini coefficients) at the network level for the heterogeneous dynamic groups. As expected, inequality increases in the dynamic networks, but remains stable in static networks. Mediation analyses show that the increased earnings of prosocials in the dynamic networks explain the effect of network structure (static or dynamic) on inequality.

## Discussion

Taken together our results show that dynamic networks promote cooperation and higher earnings, but only among prosocials. In homogeneous dynamic networks, prosocials cooperate at rates heretofore unseen in this literature. In both homogeneous and heterogeneous networks, prosocials maintain their ties longer because they cooperate more often. Greater durability of relations, in turn, leads to increased earnings. The increased earnings of prosocial actors in dynamic networks explains why these networks generate inequality, favoring prosocials.

Prior work has shown that dynamic networks promote cooperation. However, much of this work arguably conflates *network dynamics* with *reputation processes* ^7, 8, 20^. In these studies, when participants form new ties, they are given reputational information of potential alters. It is therefore unclear whether network dynamics, reputational processes^30^, or some combination of the two drives observed levels of cooperation. Here we sought to isolate the effects of pure network dynamics from reputational processes. To this end, we did not give participants any information on prospective alters’ reputations. While these reputation-free networks are obviously less realistic than networks where reputations are known, they allow us to demonstrate that network dynamics alone can promote cooperation, as long as these networks contain prosocials.

Despite the fact that prosocials in heterogeneous networks could not form new ties based on reputations or with knowledge of prospective alters’ dispositions, their relations became increasingly homophilous over time. The homogeneous networks of prosocials can thus be viewed as an exogenous imposition of how prosocials’ networks would have evolved had we allowed the initially heterogeneous networks to play out longer. That prosocials’ networks tended towards homophily and homophilous networks of prosocials cooperated at such high rates does not, however, imply that further dynamics would be unnecessary for cooperation in networks composed entirely of prosocials. Cooperation rates in homogeneous groups of prosocials were much lower in static than dynamic networks, suggesting that dynamics are also critical.

Why are dynamics needed for homogeneous networks of prosocials, despite their preference for mutual cooperation^17^? The answer, we suggest, centers on another fundamental issue confronted by persons in situations of interdependence like the PD: trust, or the expectation that alter will also cooperate^31^. Prior work shows that trust is the primary barrier to prosocials’ cooperation^32, 33^. Importantly, participants in our experiment could not communicate, nor did they have any information about why an alter might have not cooperated. Thus, especially in early rounds, a prosocial participant who defected out of concern that alter may not cooperate would likely trigger defection by the (prosocial) alter in the subsequent round. Indeed, looking only at the behaviors of prosocials, we find that cycles of defection, where a prosocial defects in response to alter's defection on the previous round, are 75% less likely to occur in dynamic networks (*p* < 0.001; *SI Appendix*), regardless of whether the networks were homogeneous or heterogeneous. With no recourse to severing ties, initially uncooperative acts in static networks likely triggered cycles of recrimination by other prosocials, leading to the lower cooperation observed in static homogeneous networks. Thus, it is not sufficient for populations to be high in prosociality: there must also be the possiblity of further dynamics.

Our findings shed light on the viability of prosocial dispositions and, more generally, the well-documented evidence of heterogeneous social preferences^14, 34, 35^. Prosocials in homogeneous and heterogeneous networks fared substantially better than their more egoistic counterparts when they could alter their networks. This suggests that the relative viability of prosocials versus egoists in dynamic networks depends on the “stickiness” of ties, or the frequency at which actors can sever or add new relations^6, 8, 20^. A key question for future work is thus how this stickiness alters the balance of population level prosociality and egoism.

## Methods

The Institutional Review Board at the University of South Carolina reviewed and approved this research, and the research was conducted in accordance with their guidelines and regulations. Informed consent was obtained from all participants before data collection. We recruited a total of 1,174 participants who completed a pre-study questionnaire with the nine-item social value orientation measure (see *SI Appendix*). Of those surveyed, 592 were classified as prosocial, 475 were classified as egoists, and the remaining 107 could not be classified. Based on social value orientations, participants were given an access code enabling them to sign up for the experiment. The access code enabled us to sort them. Participants were not informed that their survey responses affected their eligibility. On average, participants completed the pre-study survey 26.4 days (SD = 18.5) before taking part in the experiment. The data were modeled using random intercept multilevel or mixed effects models. Cooperation was fit using the logistic link with alters nested in rounds, rounds nested in participants, and participants nested in networks. Relationship duration and earnings were fit using an identity link with instances of the outcome nested in participants, and participants nested in networks. Finally, network inequality was fit using an identity link with rounds nested in networks.

## Electronic supplementary material

Supplemental Information

## References

[1] Nowak MA (2006). Five rules for the evolution of cooperation. Science.

[2] Kollock, P. Social dilemmas: The anatomy of cooperation. *Annual Review of Sociology* 183–214 (1998).

[3] Rand DG, Nowak MA (2013). Human cooperation. Trends in Cognitive Sciences.

[4] Macy, M. W. & Skvoretz, J. The evolution of trust and cooperation between strangers: A computational model. *American Sociological Review*, 638–660 (1998).

[5] Simpson B, Willer R (2015). Beyond altruism: Sociological foundations of cooperation and prosocial behavior. Annual Review of Sociology.

[6] Melamed D, Simpson B (2016). Strong ties promote the evolution of cooperation in dynamic networks. Social Networks.

[7] Rand DG, Arbesman S, Christakis NA (2011). Dynamic social networks promote cooperation in experiments with humans. Proceedings of the National Academy of Sciences.

[8] Wang J, Suri S, Watts DJ (2012). Cooperation and assortativity with dynamic partner updating. Proceedings of the National Academy of Sciences.

[9] Fehl K, van der Post DJ, Semmann D (2011). Co-evolution of behaviour and social network structure promotes human cooperation. Ecology Letters.

[10] Pacheco JM, Traulsen A, Nowak MA (2006). Coevolution of strategy and structure in complex networks with dynamical linking. Physical Review Letters.

[11] Santos FC, Pacheco JM, Lenaerts T (2006). Evolutionary dynamics of social dilemmas in structured heterogeneous populations. Proceedings of the National Academy of Sciences of the United States of America.

[12] Raub W, Weesie J (1990). Reputation and efficiency in social interactions: An example of network effects. American Journal of Sociology.

[13] Santos FC, Pacheco JM, Lenaerts T (2006). Cooperation prevails when individuals adjust their social ties. PLoS Comput Biol.

[14] Van Lange PA, De Bruin E, Otten W, Joireman JA (1997). Development of prosocial, individualistic, and competitive orientations: theory and preliminary evidence. Journal of Personality and Social Psychology.

[15] Fehr E, Gächter S (2002). Altruistic punishment in humans. Nature.

[16] Balliet D, Parks C, Joireman J (2009). Social value orientation and cooperation in social dilemmas: A meta-analysis. Group Processes & Intergroup Relations.

[17] Van Lange PA (1999). The pursuit of joint outcomes and equality in outcomes: An integrative model of social value orientation. Journal of Personality and Social Psychology.

[18] Bogaert S, Boone C, Declerck C (2008). Social value orientation and cooperation in social dilemmas: A review and conceptual model. British Journal of Social Psychology.

[19] Feinberg M, Willer R, Stellar J, Keltner D (2012). The virtues of gossip: reputational information sharing as prosocial behavior. Journal of Personality and Social Psychology.

[20] Shirado H, Fu F, Fowler JH, Christakis NA (2013). Quality versus quantity of social ties in experimental cooperative networks. Nature Communications.

[21] Axelrod, R. M. *The Evolution of Cooperation* Basic books (2006).

[22] Fischbacher U, Gächter S, Fehr E (2001). Are people conditionally cooperative? Evidence from a public goods experiment. Economics Letters.

[23] Kuhlman DM, Marshello A (1975). Individual differences in the game motives of own, relative, and joint gain. Journal of Research in Personality.

[24] Parks CD, Rumble AC (2001). Elements of reciprocity and social value orientation. Personality and Social Psychology Bulletin.

[25] Barclay P (2016). Biological markets and the effects of partner choice on cooperation and friendship. Current Opinion in Psychology.

[26] Kossinets G, Watts DJ (2009). Origins of homophily in an evolving social network1. American Journal of Sociology.

[27] Lazarsfeld PF, Merton RK (1954). Friendship as a social process: A substantive and methodological analysis. Freedom and Control in Modern Society.

[28] McPherson, M., Smith-Lovin, L. & Cook, J. M. Birds of a feather: Homophily in social networks. *Annual Review of Sociology*, 415–444 (2001).

[29] Nishi A, Shirado H, Rand DG, Christakis NA (2015). Inequality and visibility of wealth in experimental social networks. Nature.

[30] Roberts G (2015). Partner choice drives the evolution of cooperation via indirect reciprocity. PloS one.

[31] Kiyonari T, Tanida S, Yamagishi T (2000). Social exchange and reciprocity: confusion or a heuristic?. Evolution and Human Behavior.

[32] Simpson B (2004). Social values, subjective transformations, and cooperation in social dilemmas. Social Psychology Quarterly.

[33] Boone C, Declerck C, Kiyonari T (2010). Inducing cooperative behavior among proselfs versus prosocials: the moderating role of incentives and trust. Journal of Conflict Resolution..

[34] Simpson B, Willer R (2008). Altruism and indirect reciprocity: The interaction of person and situation in prosocial behavior. Social Psychology Quarterly.

[35] Emonds G, Declerck CH, Boone C, Vandervliet EJ, Parizel PM (2011). Comparing the neural basis of decision making in social dilemmas of people with different social value orientations, a fMRI study. Journal of Neuroscience, Psychology, and Economics.

