# Supplementary material for: Prosocial Orientation Alters Network Dynamics and Fosters Cooperation
Source: Sci Rep. 2017 Mar 23;7:357. doi: 10.1038/s41598-017-00265-x (PMC5427964; doi:10.1038/s41598-017-00265-x)
Supplement: Supplementary file 1 — Supplemental Information [file 41598_2017_265_MOESM1_ESM.doc]

Supplementary Information

Prosocial Orientation Alters Network Dynamics and Fosters Cooperation

David Melamed, Brent Simpson, and Ashley Harrell

1. Experimental Details

The experiment, briefly described in the main text, consisted of two different parts: a pre-study questionnaire, which we used to classify participants’ social value orientations, and the laboratory study. We describe each of these parts in more detail here.

Pre-study Questionnaire

Participants were recruited from the general student population at the University of South Carolina. Specifically, students belonging to a voluntary research participant pool, maintained by the experimental laboratory, received an email inviting them to complete a brief online survey. In exchange they would have the chance, at a later date, to complete an experiment for money. 1,174 participants completed the pre-study questionnaire.

The pre-study survey included our measure of social values, namely the nine-item *social value orientation* measure (*1-5*). Specifically, instructions asked participants to:

*Please imagine that you have been randomly paired with another person, whom we will refer to simply as the “other.” Other is someone you do not know and that you will not knowingly meet in the future. Both you and Other will be making choices by circling either the letter A, B, or C. Your own choices will produce points for yourself and Other. Likewise, Other’s choice will produce points for him/her and for you. Every point has value: The more points you receive, the better for you, and the more points Other receives, the better for him/her.*

*Here’s an example of how this task works.*

|  | *A* | *B* | *C* |
| --- | --- | --- | --- |
| *You Get* | *500* | *500* | *550* |
| *Other Gets* | *100* | *500* | *300* |

*In this example, if you chose A you would receive 500 points and Other would receive 100 points; if you chose B, you would receive 500 points and Other 500; and if you chose C, you would receive 550 points and Other 300. So, you see that your choice influences both the number of points you receive and the number of points the other receives.*

*Before you begin making choices, keep in mind that there are no right or wrong answers – choose the option that you, for whatever reason, prefer most. Also, remember that the points have value: The more of them you accumulate, the better for you. Likewise, from the Other’s point of view, the more points s/he accumulates, the better for him/her.*

*For the following questions, please choose as you see fit. Remember, there are no right or wrong answers.*

|  | *A* | *B* | *C* |
| --- | --- | --- | --- |
| *You Get* | *480* | *540* | *480* |
| *Other Gets* | *80* | *280* | *480* |

|  | *A* | *B* | *C* |
| --- | --- | --- | --- |
| *You Get* | *560* | *500* | *500* |
| *Other Gets* | *300* | *500* | *100* |

|  | *A* | *B* | *C* |
| --- | --- | --- | --- |
| *You Get* | *520* | *520* | *580* |
| *Other Gets* | *520* | *120* | *320* |

|  | *A* | *B* | *C* |
| --- | --- | --- | --- |
| *You Get* | *500* | *560* | *490* |
| *Other Gets* | *100* | *300* | *490* |

|  | *A* | *B* | *C* |
| --- | --- | --- | --- |
| *You Get* | *560* | *500* | *490* |
| *Other Gets* | *300* | *500* | *90* |

|  | *A* | *B* | *C* |
| --- | --- | --- | --- |
| *You Get* | *500* | *500* | *570* |
| *Other Gets* | *500* | *100* | *300* |

|  | *A* | *B* | *C* |
| --- | --- | --- | --- |
| *You Get* | *510* | *560* | *510* |
| *Other Gets* | *510* | *300* | *110* |

|  | *A* | *B* | *C* |
| --- | --- | --- | --- |
| *You Get* | *550* | *500* | *500* |
| *Other Gets* | *300* | *100* | *500* |

|  | *A* | *B* | *C* |
| --- | --- | --- | --- |
| *You Get* | *480* | *490* | *540* |
| *Other Gets* | *100* | *490* | *300* |

Prosocial choices are as follows: 1c, 2b, 3a, 4c, 5b, 6a, 7a, 8c, and 9b. Individualist choices are: 1b, 2a, 3c, 4b, 5a, 6c, 7b, 8a, and 9c. Competitive choices are: 1a, 2c, 3b, 4a, 5c, 6b, 7c, 8b, and 9a (*1*). As is standard in the social value orientation literature (*1, 6*), a participant was classified as a given “type” when he or she answered at least six of the nine items consistently, and were “unclassified” if not. Following past work, we combined individualists and competitors into one “egoistic” category (*1,4,6*).

Of those who completed the survey, 592 participants (50.4%) could be classified as prosocial (mean number of prosocial responses = 8.52, SD = 0.91) and 475 (40.4%) could be classified as egoist (mean number of prosocial responses = 0.58, SD = 1.03). The remaining 107 participants could not be classified.

A few days after completing the survey, participants who could be classified as a given social value type were given an access code, which allowed them to sign up for the laboratory experiment. The access code differed for those categorized as prosocial and those categorized as egoist; this allowed the researcher to schedule study sessions based on participants’ social value orientations, as described below. At no point were participants told that their responses to the survey had determined their eligibility for the experiment. On average, participants completed the pre-study survey 26.4 days (SD= 18.5) before taking part in the experiment, which is described next.

Laboratory Experiment

Participants in the experiment were recruited from among those who had completed the pre-study questionnaire and had been classified as either prosocial or egoist. A total of 360 participants (30 groups of 12 participants each, 75.3% female) completed the experiment, in exchange for the opportunity to earn money. Sessions were scheduled such that 10 groups were *heterogeneous* (50% prosocial and 50% egoist),[[1]](#footnote-2) 10 were *homogeneous-prosocial* (100% prosocial), and the remaining 10 were *homogeneous-egoist* (100% egoist). All groups completed both a *static* and *dynamic* network phase, as described in more detail below.

As participants arrived to the laboratory, they were seated at an isolated computer station where they completed a consent form. Once all 12 participants were ready to begin, everyone was able to proceed to the study instructions. The entire study took part over a computer network, and was programmed in z-Tree version 3.4.2 (*7*).

Instructions first assured participants of their anonymity during the experiment. Specifically, participants were told that while they would be interacting with some or all of 11 others during the study session, all interactions with the others would take place over the computer. Next were the instructions for a standard Prisoner’s Dilemma (PD). These were presented over a series of screens, and are reproduced below:

*The study today consists of several different parts.*

*In* ***Part 1*** *of the study, you will initially be connected to* ***3*** *of the 11 other participants. You will make decisions with each of these 3 others over the computer.*

***Specifically:*** *for* ***each*** *of the 3 others with whom you are connected, you will be asked whether you want to contribute to a* ***joint fund*** *with that other. At the same time, each of the three others will be deciding whether they want to contribute to the joint fund with each of the people they are connected with, including you.*

***Importantly, how much you earn from the joint fund depends on the choices that you and the other have made. Specifically:***

*If* ***you both contribute*** *to the joint fund,* ***you both earn 4 points****.*

*If* ***you contribute*** *to the joint fund, but* ***the other participant does not****,* ***you lose 1 point*** *and* ***the other participant gains 7 points.***

*If* ***you do not contribute*** *to the joint fund, but* ***the other participant does, you gain 7 points*** *and* ***the other participant loses 1 point****.*

*If* ***you both do not contribute*** *to the joint fund,* ***you both earn 1 point.***

*(This information is also on the handout on your desk, which you can refer back to during the study, if you need to.)*

*You will make decisions to contribute to the joint fund in each of several different rounds. At the end of the study, you will be paid based on your total earnings over each round.*

The experiment consisted of two different phases of the repeated PD: the *static* phase and the *dynamic* phase. Groups completed the two phases in random order, and this order determined which set of instructions was presented next. For the static phase [dynamic phase in brackets]:

*After you decide whether to contribute to the joint fund with each of the others, you will see whether each of the others decided to contribute to the joint fund with you. You will also see both your earnings and the others' earnings for the round.*

***Importantly, when the next round begins, you will [may not] be paired with the same others. That is, the others with whom you are making decisions will not [may] change in subsequent rounds.***

*[Specifically, you will be asked whether you want to* ***stop interacting with one of the participants with whom you are connected, and start interacting with a new participant.*** *If you do, you will choose which one other you no longer wish to interact with, and then you will be linked at random with a new participant. While you are deciding whether and with whom you wish to stop interacting,* ***the others will be deciding whether they wish to continue interacting with their ties, including you.*** *(As a result, you may be tied to fewer than three others, or possibly no others, in some rounds.)]*

*[****Note that,*** *if you choose to stop interacting with a participant,* ***or*** *if they choose to stop interacting with you,* ***you will not interact with that participant again throughout the remaining rounds of Part 1.****]*

*Once everyone has made their decisions and viewed the results, a new round will begin. You will again decide whether or not to contribute to the joint fund with the same interaction partners that you were connected with in the previous round [the interaction partners that you chose to remain connected with* and *who chose to remain connected with you, plus any new others with whom you have been connected]. This process will continue for several rounds.*

Next, participants completed several comprehension questions to ensure their understanding of the payoff structure of the PD, and how it varied based on their and the others’ choices. The vast majority of participants were able to answer the comprehension questions correctly. Any incorrect responses were followed by a detailed explanation of the correct answer.

Once everyone had completed the comprehension check, the PD task began. At the start of each round, each participant could see a list of his or her alters, identified by a unique letter. They could also see the results of their interaction with each alter in the previous round (except at round one or, in the dynamic condition, if the alter was a new tie). Instructions prompted participants to make their decisions to cooperate or not with each alter:

*Below is a list of each other participant with whom you are connected. You can also see whether the other participant contributed to the joint fund with you in the last round, if applicable. For* ***each Other ID below****,*

*select* ***Yes if you* would *like to contribute to the joint fund with that other,*** *or,*

*select* ***No if you* would *not like to contribute to the joint fund with that other****.*

*You may use the handout on your desk if you need a reminder of how your and the other's decisions will impact your and the other's earnings.*

*When you have finished making your decisions, click Continue.*

Once everyone had made their decisions, a results screen listed the participant’s and each of his or her alters’ decisions and earnings for the round, as well as the participant’s total earnings across all alters for the round. In the static phase, the study then progressed to the next round, and this process was repeated for 12 rounds total. Participants did not know in advance the number of rounds they would complete.

In the dynamic phase, after viewing the results, participants viewed the same screen again, but this time they were prompted to indicate whether or not they wished to stop interacting with one of the others to whom they were tied. If so, they could select one alter that they wished to drop, and would be randomly tied to a new alter. Once each participant had made their decisions, they could view a final screen which listed who, if anyone, they had stopped interacting with; who, if anyone, had stopped interacting with them, and who, if anyone, they had picked up as a new tie. As in the static phase, this process was repeated for 12 rounds.

After the 12 rounds of the first phase (either static or dynamic, depending on random order) were completed, participants were told that they would move on to Part 2 of the study. Identifying letters were changed from Part 1 to Part 2; participants were informed that they and each of the others had received a new identifying letter.

Part 2, according to the instructions, would be similar to Part 1—each person would be initially tied to three randomly selected others, and would make decisions with those others to contribute or not to the “joint fund”, following the same payoff structure from Part 1. Participants who had completed the dynamic phase first then read the instructions for the static phase; likewise, groups that had already completed the static phase completed instructions for the dynamic phase. After another manipulation check quiz question to check their understanding of the new phase, the study proceeded as described above, for another 12 rounds.

After both phases were completed, participants were probed for suspicion, paid based on their earnings over the 24 rounds of the study (earnings ranged from $10 to $15), and debriefed. The study took approximately 45 minutes to complete.

2 Statistical Analyses

Tables S1 and S2 present the descriptive statistics for the dynamic and static networks, respectively. All of these values aggregate over the 12 rounds. Figures 1-4 present the marginal means through time. In both dynamic and static networks, prosocials in homogeneous networks cooperated the most, then prosocials in heterogeneous networks, then egoists in heterogeneous networks, and egoists in homogeneous networks cooperated the least. In the heterogeneous dynamic networks, prosocials’ networks became more homophilous, on average, than egoists’ networks. In the dynamic networks, prosocials in heterogeneous networks acquired the most ties, while egoists in homogeneous networks acquired the fewest.

In terms of earnings (fitness), the ordering was again the same in both dynamic and static networks. Prosocials in homogeneous networks earned the most, then egoists in heterogeneous networks, then prosocials in heterogeneous networks, and finally egoists in homogeneous networks earned the least.

Table S1: Descriptive Statistics for Dynamic Networks

|  | Prosocials | | Egoists | |
| --- | --- | --- | --- | --- |
|  | Homogeneous | Heterogeneous | Homogeneous | Heterogeneous |
| Cooperate | .76 | .65 | .45 | .51 |
| Duration | 4.53  (3.95) | 5.01  (3.90) | 3.85  (3.21) | 3.89  (3.35) |
| Homophily |  | .53 |  | .40 |
| Number of Ties | 3.04  (1.16) | 3.31  (1.21) | 2.91  (1.08) | 2.76  (1.08) |
| Earnings | 3.40  (2.17) | 2.88  (2.41) | 2.51  (2.51) | 2.98  (2.59) |

Table S2: Descriptive Statistics for Static Networks

|  | Prosocials | | Egoists | |
| --- | --- | --- | --- | --- |
|  | Homogeneous | Heterogeneous | Homogeneous | Heterogeneous |
| Cooperate | .56 | .47 | .32 | .38 |
| Earnings | 2.79  (2.32) | 2.37  (2.24) | 2.11  (2.51) | 2.43  (2.45) |

Table S3 presents the results of five random intercept generalized linear mixed models predicting cooperation with each alter, with alters nested in rounds, rounds nested in participants, and participants nested in networks. All of the models include round, round-squared, and round-cubed. Likelihood ratio tests of nested models were used to determine the specification of rounds. Model 1 includes controls for whether the participant completed the dynamic network first, whether the participant was male, and whether alter cooperated on the previous round. Of the controls, only whether alter cooperated on the previous round is significant. However, including lagged cooperation of alters reduces the sample size since participants did not interact with all alters on the previous round. Removing lagged cooperation of alters (model 2) does not change the substantive conclusions for the main actor type and network type effects. The only significant change is that time becomes significant when lagged cooperation of alters is constrained. Because of the reduced sample size and the fact that substantive conclusions remain the same, in subsequent models we remove the effect of lagged cooperation of alters, along with the other insignificant controls. In all subsequent models, cooperation declines quickly and then recovers slightly, and tails off again towards the end of the study. Neither of these was significant, so they were dropped from subsequent models. Models 3-5 only differ in the reference category. This was done to determine significant contrasts. The italicized coefficients in Table S3 are reported in the main text.

Table S3: Results from 4-level generalized linear mixed models predicting cooperation

|  | | Model 1 | Model 2 | Model 3 | Model 4 | Model 5 |
| --- | --- | --- | --- | --- | --- | --- |
| Egoists | |  |  |  |  |  |
|  | Static & Homogeneous^1^ | -2.387  (.367) | .775^*^  (.307) | .815^**^  (.286) | -1.564^***^  (.380) | -.947^*^  (.416) |
|  | Static & Heterogeneous | .026  (.363) | .353  (.417) | .357  (.418) | -1.206^**^  (.418) | -.590  (.346) |
|  | Dynamic & Homogeneous | .327  (.318) | .647  (.378) | *.645*  *(.380)* | -.919^*^  (.380) | -.302  (.416) |
|  | Dynamic & Heterogeneous | .429  (.370) | .954^*^  (.418) | *.960^*^*  *(.420)* | -.604  (.420) | .013  (.453) |
| Prosocials | |  |  |  |  |  |
|  | Static & Homogeneous^2^ | 1.167^***^  (.316) | 1.556^***^  (.378) | 1.564^***^  (.380) | 2.379^***^  (.287) | .617  (.416) |
|  | Static & Heterogeneous^3^ | .824^*^  (.359) | .957^*^  (.414) | .947^*^  (.416) | -.617  (.416) | 1.762^***^  (.333) |
|  | Dynamic & Homogeneous | 2.029^***^  (.323) | 2.538^***^ (.380) | 2.545^***^  (.382) | *.981^**^*  *(.382)* | 1.598^***^  (.418) |
|  | Dynamic & Heterogeneous | 1.522^***^  (.364) | 1.964^***^  (.417) | 1.955^***^  (.418) | .392  (.418) | *1.008^*^*  *(.451)* |
| Control Variables | |  |  |  |  |  |
|  | Round (R) | -.189  (.148) | -.911^***^  (.064) | -.911^***^  (.064) | -.911^***^  (.064) | -.911^***^  (.064) |
|  | R x R | .026  (.023) | .119^***^  (.011) | .119^***^  (.011) | .119^***^  (.011) | .119^***^  (.011) |
|  | R x R x R | -.001  (.001) | -.005^***^  (.001) | -.005^***^  (.001) | -.005^***^  (.001) | -.005^***^  (.001) |
|  | Dynamic First (=1) | -.096  (.185) | -.037  (.220) |  |  |  |
|  | Male Participant | .256  (.173) | .247  (.169) |  |  |  |
|  | Alter Cooperated Last Round | 3.343^***^  (.070) |  |  |  |  |
| Variance Components | |  |  |  |  |  |
|  | Round | 1.183^***^  (.109) | .196^***^  (.039) | .196^***^  (.039) | .196^***^  (.039) | .196^***^  (.039) |
|  | Participant | 3.139^***^  (.256) | 3.275^***^  (.233) | 3.286^***^  (.234) | 3.286^***^  (.234) | 3.286^***^  (.234) |
|  | Network | .189^*^  (.096) | .413^**^  (.132) | .421^**^  (.134) | .421^**^  (.134) | .421^**^  (.134) |

*Note*: ^*^*p* < .05, ^**^*p* < .01, ^***^*p* < .001. These are the same models, but the reference category was changed to determine significant contrasts. ^1^Reference category for models one, two and three, ^2^reference category for model four, and ^3^reference category for model five. Egoist/prosocial contrasts in the column are in terms of deviations from those cells. Model 1, N = 21,572 network-participant-round-alters. Models 2-5, N = 25,630 network-participant-round-alters.

Table S4 illustrates how the effects of network type and social values vary with time. A test of nested models demonstrated that these factors do indeed vary over the rounds in the experiment ($\chi_{(14)}^{2}=94.07$, *p* < .001). The margins from this model were used to generate Figure 1. For all marginal means in the Figures, delta method standard errors were used to determine standard errors around the point estimates (*8-9*). For the predicted probabilities, point estimates and standard errors were obtained from the linear predictors, and the logistic function was used to transform the linear predictors into predicted probabilities.

Table S4: Results from 4-level generalized linear mixed models predicting cooperation with interactions through time

|  | | Round (R) | R x R | R x R x R |
| --- | --- | --- | --- | --- |
| Egoists | |  |  |  |
|  | Static & Homogeneous | -1.184^***^  (.130) | .165^***^  (.023) | -.007^***^  (.001) |
|  | Static & Heterogeneous | -1.021^***^  (.163) | .109^***^  (.030) | -.004^*^  (.002) |
|  | Dynamic & Homogeneous | -1.027^***^  (.130) | .135^***^  (.023) | -.005^***^  (.001) |
|  | Dynamic & Heterogeneous | -1.220^***^  (.170) | .189^***^  (.032) | -.009^***^  (.002) |
| Prosocials | |  |  |  |
|  | Static & Homogeneous | -.889^***^  (.134) | .107^***^  (.023) | -.004^***^  (.001) |
|  | Static & Heterogeneous | -.957^***^  (.159) | .118^***^  (.029) | -.005^**^  (.002) |
|  | Dynamic & Homogeneous | -.217  (.142) | .023  (.026) | -.001  (.001) |
|  | Dynamic & Heterogeneous | -.702^***^  (.167) | .095^**^  (.031) | -.004^**^  (.002) |
|  |  |  |  |  |
| Constant | |  |  | 1.877^***^  (.157) |
| Variance Components | |  |  |  |
|  | Round |  |  | .185^***^  (.038) |
|  | Participant |  |  | 3.247^***^  (.232) |
|  | Network |  |  | .593^***^  (.179) |

*Note*: ^**^*p* < .01, ^***^*p* < .001. This is one single model. Marginal probabilities from this model were used to generate Figure 1. N = 25,630 network-participant-round-alters.

Table S5 presents the results from a conditional logistic regression predicting which alter was dropped when participants in the dynamic networks dropped an alter. The variance/covariance matrix was clustered on networks to adjust for the nested data structure; similar results are obtained when we cluster on participants. The margins from this model are reported in the main text. In general, those who cooperated on the previous round were less likely to be dropped, as were prosocial alters. A second model (not shown) demonstrates that alters who cooperated two rounds ago are also less likely to be dropped (b = -.97, *p* < .001), but the other results hold.

Table S5: Conditional logistic predicting which alter is dropped when participants chose to delete a tie in dynamic networks.

| Alter Cooperated on the Previous Round (=1) | -1.44^***^  (.26) |
| --- | --- |
| Alter is Prosocial (=1) | -.81^***^  (.13) |

*Note*: ^***^*p* < .001. N = 1,648 participant-choices. Standard errors were adjusted for nesting at the network level.

Table S6 presents three random intercept linear mixed models predicting relationship duration, with alters nested in participants, and participants nested in networks. Here the outcome is how long ego was connected to each alter in the dynamic networks. Model 1 includes controls for whether the participant completed the dynamic network condition first, whether the participant was male, and whether the network was heterogeneous (vs. homogeneous). None of these controls were significant so they were dropped from subsequent models. Model 2 shows that prosocial participants kept their alters, on average, .972 additional rounds. Model 3 shows that including the number of times that ego cooperated with alter explains the effect of prosociality on duration. When this is included in the model, prosociality becomes insignificant. For each additional time that ego cooperated with alter, on average, ego remains connected to alter for .909 additional rounds.

Table S6: Results from 3-level linear mixed models predicting duration in the dynamic networks

|  |  | Model 1 | Model 2 | Model 3 |
| --- | --- | --- | --- | --- |
| Prosocial (=1) | | .960^***^  (.214) | .972^***^  (.217) | -.262  (.141) |
| Cooperation with Alter | |  |  | .909^***^  (.011) |
| Heterogeneous Network (=1) | | .383  (.314) |  |  |
| Dynamic First (=1) | | -.347  (.296) |  |  |
| Male Participant (=1) | | -.068  (.181) |  |  |
| Constant | | 3.992^***^  (.261) | 3.910^***^  (.187) | 2.062^***^  (.130) |
| Variance Components | |  |  |  |
|  | Participant | .788^***^  (.112) | .788^***^  (.112) | .627^***^  (.053) |
|  | Network | .687^***^  (.126) | .725^***^  (.130) | .528^***^  (.090) |

*Note:* ^***^*p* < .001. N = 2,978 network-participants-alters.

Table S7 presents two random intercept linear mixed models predicting homophily in the heterogeneous dynamic networks, with rounds nested in participants, and participants nested in networks. The outcome is the proportion of ego’s network that is homophilous. Model 1 demonstrates that the control variables are insignificant, and Model 2 is the model that is discussed in the main text.

Table S7: Results from 3-level linear mixed models predicting proportion of SVO homophily in heterogeneous dynamic networks.

|  | | Model 1 | Model 2 |
| --- | --- | --- | --- |
| Prosocials (P) | | .050  (.039) | .052  (.039) |
| Round (R) | | .002  (.003) | .002  (.003) |
| P x R | | .013^***^  (.002) | .013^***^  (.002) |
| Constant | | .382^***^  (.041) | .393^***^  (.031) |
| Control Variables | |  |  |
|  | Dynamic First (=1) | .035  (.042) |  |
|  | Male Participant | -.038  (.037) |  |
| Variance Components | |  |  |
|  | Participant | .156^***^  (.013) | .157^***^  (.013) |
|  | Network | .042  (.023) | .045^*^  (.022) |

*Note*: ^***^*p* < .001. N = 1,411 network-participant-rounds. Linear predictors from Model 2 were used to generate Figure 2a.

Table S8 presents two random intercept linear mixed models predicting the number of ties in the dynamic networks, with rounds nested in participants, and participants nested in networks. Again, Model 1 demonstrates that the controls are insignificant, and Model 2 is the model that is discussed in the main text. It is worth noting that all participants (egoists and prosocials) began the experiment with three ties. Thus, while it may appear in Fig. 2B as if prosocials had more ties, on average, than egoists in round 1, the figure gives marginal means from Model 2 in S8, not the observed data.

Table S8: Results from 3-level linear mixed models predicting number of ties in heterogeneous dynamic networks

|  | | Model 1 | Model 2 |
| --- | --- | --- | --- |
| Prosocials (P) | | .145  (.176) | .143  (.176) |
| Round (R) | | -.025^**^  (.009) | -.025^**^  (.009) |
| P x R | | .040^***^  (.009) | .040^***^  (.009) |
| Constant | |  | 2.895^***^  (.125) |
| Control Variables | |  |  |
|  | Dynamic First (=1) | .069  (.160) |  |
|  | Male Participant | .002  (.184) |  |
| Variance Components | |  |  |
|  | Participant | .828^***^  (.058) | .829^***^  (.061) |
|  | Network | .000  (.000) | .000  (.000) |

*Note*: ^**^*p* < .01, ^***^*p* < .001. N = 1,411 network-participant-rounds. Linear predictors from Model 2 were used to generate Figure 2b.

Table S9 presents four random intercept linear mixed models predicting total earnings for the round, with rounds nested in participants, and participants nested in networks. Likelihood ratio tests of nested models were again used to specify the effect of rounds on earnings. Model 1 illustrates that the controls are insignificant. Models 2-4 are the same model with different reference categories. Model 2 demonstrates that prosocials earn significantly more in dynamic networks (only heterogeneous shown). Model 3 demonstrates that egoists in heterogeneous dynamic networks do not earn more than egoists in heterogeneous static networks, and Model 4 demonstrates that egoists in homogeneous dynamic networks do not earn more than egoists in homogeneous static networks.

Table S9: Results from 3-level linear mixed models predicting earnings

|  | | Model 1 | Model 2 | Model 3 | Model 4 |
| --- | --- | --- | --- | --- | --- |
| Egoists | |  |  |  |  |
|  | Static & Homogeneous^3^ | -3.175  (.608) | -3.159^***^  (.609) | -.975  (.611) | 7.986^***^  (.431) |
|  | Static & Heterogeneous^2^ | -2.185^***^  (.651) | -2.183^***^  (.653) | 8.961^***^  (.491) | .975  (.611) |
|  | Dynamic & Homogeneous | -2.205^***^  (.608) | -2.189^***^  (.609) | -.005  (.611) | *.970*  *(.564)* |
|  | Dynamic & Heterogeneous | -1.299^**^  (.465) | -1.298^**^  (.465) | *.886*  *(.656)* | 1.861^**^  (.612) |
| Prosocials | |  |  |  |  |
|  | Static & Homogeneous | -1.137  (.608) | -1.120  (.609) | 1.063  (.611) | 2.039^***^  (.564) |
|  | Static & Heterogeneous | -2.354^***^  (.649) | *-2.354^***^*  *(.650)* | -.170  (.463) | .805  (.608) |
|  | Dynamic & Homogeneous | .783  (.609) | .801  (.609) | 2.983^***^  (.612) | 3.959^***^  (.565) |
|  | Dynamic & Heterogeneous^1^ | 11.229^***^  (.527) | 11.145^***^  (.488) | 2.183^***^  (.653) | 3.159^***^ (.609) |
| Control Variables | |  |  |  |  |
|  | Round (R) | -.529^***^  (.060) | -.529^***^  (.060) | -.529^***^  (.060) | -.529^***^  (.060) |
|  | R x R | .033^***^  (.005) | .033^***^  (.005) | .033^***^  (.005) | .033^***^  (.005) |
|  | Dynamic First (=1) | -0.157  (.327) |  |  |  |
|  | Male Participant | .043  (.226) |  |  |  |
| Variance Components | |  |  |  |  |
|  | Participant | 2.191^***^  (.082) | 2.191^***^  (.082) | 2.191^***^  (.082) | 2.191^***^  (.082) |
|  | Network | 1.023^***^  (.142) | 1.028^***^  (.142) | 1.028^***^  (.142) | 1.028^***^  (.142) |

*Note*: ^**^*p* < .01, ^***^*p* < .001. ^1^Reference category for Models 1 and 2, ^2^reference category for Model 3, and ^3^reference category for Model 4; egoist/prosocial contrasts in each column are in terms of deviations from these rows. N = 8,545 network-participant-rounds.

Table S10 presents three more random intercept linear mixed models predicting total earnings for the round, with the same nesting structure as above. Model 1 shows that when relationship duration, interacted with social values and network type, is included in the model, prosocials in dynamic heterogeneous networks no longer earn significantly more than prosocials in static heterogeneous networks. Similarly, Model 2 alters the reference category to demonstrate that when duration is included, prosocials in dynamic homogeneous networks no longer earn significantly more than prosocials in static homogeneous networks. That is, the positive effect of relationship duration for prosocials in dynamic networks explains why they earn more in both homogeneous and heterogeneous dynamic networks. Model 3 shows that the effect of dynamic networks on the earnings of prosocials is still significant when the number of times that ego cooperated with alter is included in the model. The effect of the number of times that ego cooperated with alter does not vary with social values and network type ($\chi_{(3)}^{2}=1.99$, *p* = n.s.). So, cooperation explains why prosocials keep alters longer, but it does not explain why prosocials earn more. Together, the results so far suggest that prosocials cooperate more, which leads them to maintain their relationships. These longer relationships explain why they earn more in dynamic networks. Model 3 illustrates that we can’t simplify this to say that cooperation explains earnings: cooperation operates through duration. Table S11 presents the final mixed model for earnings. This model allows the effects of social values and network type to vary with time. The marginal means from this model were used to generate Figure 3.

Table S10: Results from 3-level linear mixed models predicting earnings

|  | | Model 1 | Model 2 | Model 3 |
| --- | --- | --- | --- | --- |
| Egoists | |  |  |  |
|  | Static & Homogeneous | -.549  (.648) | -1.782^**^  (.608) | -1.742^**^  (.509) |
|  | Static & Heterogeneous | .427  (.519) | -.807  (.650) | -.832  (.553) |
|  | Dynamic & Homogeneous | -.252  (.691) | -1.486^*^  (.610) | -.693  (.510) |
|  | Dynamic & Heterogeneous | .636  (.691) | -.598  (.654) | .129  (.555) |
| Prosocials | |  |  |  |
|  | Static & Homogeneous^2^ | 1.234^*^  (.597) | 10.491^***^  (.451) | 9.350^***^  (.015) |
|  | Static & Heterogeneous^1^ | 9.258^***^  (.503) | -1.234^*^  (.597) | -1.123^*^  (.550) |
|  | Dynamic & Homogeneous | 1.232  (.648) | -.002  (.608) | 2.008^***^  (.508) |
|  | Dynamic & Heterogeneous | .412  (.685) | -.822  (.648) | 1.264^*^  (.550) |
| Duration | |  |  |  |
|  | Egoist & Static | -.099^*^  (.042) | -.099^*^  (.042) |  |
|  | Egoist & Dynamic | .107  (.057) | .107  (.057) |  |
|  | Prosocial & Static | .039  (.039) | .039  (.039) |  |
|  | Prosocial & Dynamic | .458^***^  (.049) | .458^***^  (.049) |  |
| Cooperation with Alter | |  |  | .105^***^  (.015) |
| Control Variables | |  |  |  |
|  | Round (R) | -.542^***^  (.067) | -.542^***^  (.067) | -.541^***^  (.061) |
|  | R x R | .033^***^  (.005) | .033^***^  (.005) | .034^***^  (.005) |
| Variance Components | |  |  |  |
|  | Participant | 2.138^***^  (.082) | 2.138^***^  (.082) | 1.984^***^  (.084) |
|  | Network | 1.008^***^  (.140) | 1.008^***^  (.140) | .905^***^  (.132) |

*Note*: ^*^*p* < .05, ^**^*p* < .01, ^***^*p* < .001. These are the same models, but the reference category was changed to determine significant contrasts. ^1^Reference category for model 1, ^2^Reference category for models 2 and 3; egoist/prosocial contrasts in each column are in terms of deviations from this cell. N = 8,545 network-participant-rounds.

Table S11: Results from 3-level linear mixed models predicting earnings

|  | | Round (R) | R x R |
| --- | --- | --- | --- |
| Egoists | |  |  |
|  | Static & Homogeneous | -.864^***^  (.118) | .052^***^  (.009) |
|  | Static & Heterogeneous | -.751^***^  (.147) | .036^**^  (.012) |
|  | Dynamic & Homogeneous | -.688^***^  (.119) | .044^***^  (.009) |
|  | Dynamic & Heterogeneous | -.440^**^  (.148) | .020  (.012) |
| Prosocials | |  |  |
|  | Static & Homogeneous | -.527^***^  (.118) | .028^**^  (.009) |
|  | Static & Heterogeneous | -.746^***^  (.145) | .043^***^  (.012) |
|  | Dynamic & Homogeneous | -.042  (.119) | .012  (.009) |
|  | Dynamic & Heterogeneous^1^ | -.182  (.146) | .022  (.012) |
| Variance Components | |  |  |
|  | Participant |  | 2.200^***^  (.082) |
|  | Network |  | 1.095^***^  (.152) |

*Note*: ^**^*p* < .01, ^***^*p* < .001. This is one single model. Marginal means from this model were used to generate Figure 3. N = 8,545 network-participant-rounds.

Table S12 presents two random intercept linear mixed models predicting network-level inequality (Gini coefficients), with rounds nested in networks. There were 240 networks: 10 heterogeneous static networks and 10 heterogeneous dynamic networks, each with 12 rounds. Model 1, which was used to generate Figure 4, shows that inequality increases in the dynamic networks, but not in the static networks. Model 2 shows that this effect goes to zero when the earnings of prosocials are included in the model. Thus, the increased earnings of prosocials in the dynamic networks increases inequality at the network level. A logical chain is implied by our results: prosocials cooperate more, which leads them to keep ties longer. In turn, durable relations explain why they earn more in dynamic networks. Because they earn more in dynamic networks, dynamic networks show an increase in inequality through time, favoring prosocials.

Table S12: Results from 2-level linear mixed models predicting inequality in heterogeneous networks

|  | | Model 1 | Model 2 |
| --- | --- | --- | --- |
| Round (R) | | .000  (.000) | -.009  (.005) |
| Dynamic Network (D) | | .006  (.015) | .017  (.015) |
| R x D | | .005^**^  (.002) | .003  (.002) |
| Prosocial Earnings (P) | |  | -.007^*^  (.003) |
| R x P | |  | .001^*^  (.000) |
| Constant | | .198^***^  (.010) | .276^***^  (.038) |
| Variance Component | |  |  |
|  | Network | .018^***^  (.004) | .016^***^  (.005) |

*Note*: ^*^*p* < .05, ^**^*p* < .01, ^***^*p* < .001. Marginal means from Model 1 were used to generate Figure 4. N = 240 network-rounds

Finally, Table S13 presents four random intercept generalized linear mixed models predicting cycles of defection, with alters nested in rounds, rounds nested in participants, and participants nested in networks. Models 1 and 2 were estimated on the behaviors of only prosocial actors, while Models 3 and 4 were estimated on the behaviors of both prosocials and egoists. Model 1 shows that cycles of defection are 75% less likely to occur in dynamic networks than in static ones (i.e., exp(-1.387) = .25). Model 2 shows that the effect of dynamic networks on cycles of defection does not vary by whether the prosocial actors are in homogeneous or heterogeneous networks. Models 3 and 4 are shown to demonstrate how cycles of defection operate among egoists. Model 3 replicates the previous finding that cycles of defection are less likely in dynamic networks. Model 3 also shows that egoists are more likely, on average, to be in a cycle of defection. Model 4 includes all 2-way interaction effects. Importantly, dynamic networks do not interact with the other factors, meaning that cycles of defection are less likely in dynamic networks regardless of whether the network is heterogeneous or whether the participant is prosocial. There is a significant interaction between prosocials and heterogeneous networks showing that prosocials are more likely to be in cycles of defection in heterogeneous networks (i.e., when they interact with egoists).

Table S13: Results from 4-level generalized linear mixed models predicting cycles of defection (=1 if ego defected and alter defected on the previous round).

|  | | Model 1 | Model 2 | Model 3 | Model 4 |
| --- | --- | --- | --- | --- | --- |
| Dynamic (D) | | -1.387^***^  (.299) | -1.556^***^  (.404) | -1.119^***^  (.228) | -.975^**^  (.282) |
| Heterogeneous (H) | | .866^**^  (.300) | .684  (.417) | .194  (.241) | -.471  (.334) |
| Prosocial (P) | |  |  | -.579^***^  (.153) | -1.388^***^  (.282) |
| D $\times$ H | |  | -.502  (.421) |  | .036  (.426) |
| D $\times$ P | |  |  |  | -.314  (.278) |
| P $\times$ H | |  |  |  | 1.303^***^  (.298) |
| Constant | | -.896^***^  (.250) | -.813^**^  (.282) | .075  (.194) | .483^*^  (.223) |
| Variance Components | |  |  |  |  |
|  | Round | .000  (.000) | .000  (.000) | .013  (.033) | .013  (.033) |
|  | Participant | 1.760^***^  (.191) | 1.761^***^  (.191) | 1.401^***^  (.105) | 1.390^***^  (.104) |
|  | Network | .634^**^  (.203) | .625^**^  (.201) | .634^***^  (.153) | .465^***^  (.113) |

*Note:* ^*^*p* < .05, ^**^*p* < .01, ^***^*p* < .001. Models 1-2, N = 11,189 network-participant-round-alters. Models 3-4, N = 21,572 network-participant-round-alters.

References

1. Van Lange, P.A., De Bruin, E., Otten, W. & Joireman, J.A. Development of prosocial, individualistic, and competitive orientations: theory and preliminary evidence. *Journal of Personality and Social Psychology* 73(4), 733 (1997).

2. Balliet, D., Parks, C., & Joireman, J. Social value orientation and cooperation in social dilemmas: A meta-analysis. *Group Processes & Intergroup Relations* 12(4), 533–547 (2009).

3. Bogaert, S., Boone, C. & Declerck, C. Social value orientation and cooperation in social dilemmas: A review and conceptual model. *British Journal of Social Psychology* 47(3), 453–480 (2008).

4. Harrell, A. & Simpson, B. The Dynamics of Prosocial Leadership: Power and Influence in Collective Action Groups. *Social Forces* 94(3), 1283 (2015).

5. Van Prooijen, J. W., et al. The egocentric nature of procedural justice: Social value orientation as moderator of reactions to decision-making procedures. *Journal of Experimental Social Psychology* 44(5), 1303–1315 (2008).

6. Simpson, B. & Willer, R. Altruism and indirect reciprocity: The interaction of person and situation in prosocial behavior. *Social Psychology Quarterly* 71(1), 37–52 (2008).

7. Fischbacher, U. z-Tree: Zurich toolbox for ready-made economic experiments. *Experimental Economics* 10(2), 171–178 (2007).

8. Oehlert, G. W. A note on the delta method. *The American Statistician* 46(1), 27–29 (1992).

9. Rice, J. *Mathematical Statistics and Data Analysis* Nelson Education (2006).

1. One of the heterogeneous groups contained 7 prosocials and 5 egoists, instead of a 50-50 split. [↑](#footnote-ref-2)
